# ﻿Revision of *Gymnoscirtetes* (Orthoptera, Acrididae, Melanoplinae): a genus endemic to the grasslands of the southeastern North American Coastal Plain

**DOI:** 10.3897/zookeys.1134.94984

**Published:** 2022-12-07

**Authors:** JoVonn G. Hill

**Affiliations:** 1 Mississippi Entomological Museum, Department of Biochemistry, Molecular Biology, Entomology, and Plant Pathology, Mississippi State University, Mississippi, USA Mississippi State University Mississippi United States of America

**Keywords:** Alabama, biodiversity hotspot, bog, Florida, Georgia, grasshopper

## Abstract

*Gymnoscirtetes* is endemic to the southeastern portion of the North American Coastal Plain and previously comprised two species: *G.pusillus* Scudder, 1897 and *G.morsei* Hebard, 1918. Here, this genus is revised based on male genital morphology and geographic data, and four new species are described: *G.georgiaensis***sp. nov.**, *G.pageae***sp. nov.**, *G.rex***sp. nov.**, and *G.wadeorum***sp. nov.***Gymnoscirtetes* is primarily associated with mesic grasslands such as pitcher plant bogs, flatwoods, and the edges of seasonal ponds, but can be found less commonly in a variety of other grasslands.

## ﻿Introduction

The North American Coastal Plain was recently designated as the world’s 36^th^ global biodiversity hotspot based on the high levels of biodiversity and endemism of vascular plants and habitat loss greater than 70% in the region (Noss 2016). A disproportionate amount of this biodiversity is found in the imperiled grasslands of the region, though they have historically received much less attention from conservation and natural resource agencies than forests and wetlands in the region (Noss 2013; Noss et al. 2015, 2021; Hill and Barone 2018). As functionally dominant herbivores in temperate grassland systems, it stands to reason that grasshopper diversity and endemism would also be high in the region. Indeed, Hill (2018) surveyed the grasshoppers of the southeastern United States and documented 173 species (82% of the fauna) that occur in grasslands and of these 111 species (53% of the fauna) and five genera (*Aptenopedes*, *Eotettix*, *Floridacris*, *Floritettix* and, *Gymnoscirtetes*) are endemic to the region.

**Figure 1.**
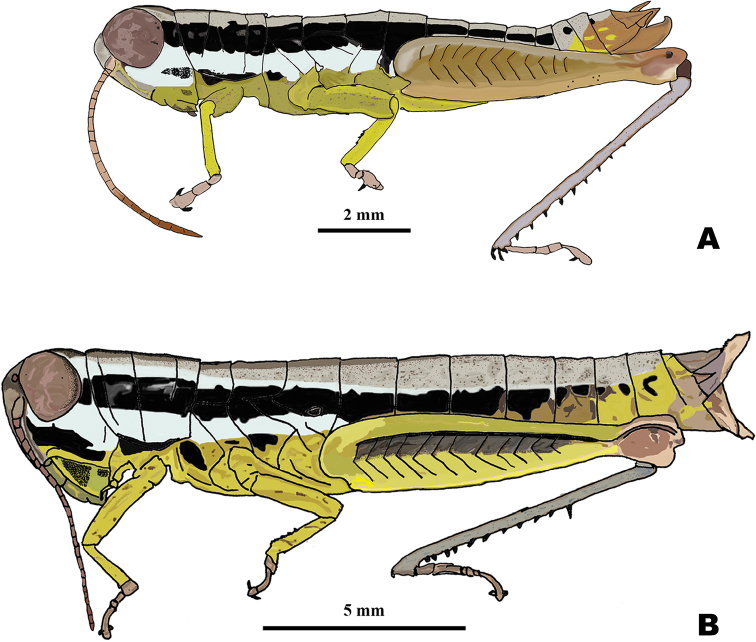
Habitus drawings of *Gymnoscirtetes***A** male **B** female.

**Figure 2.**
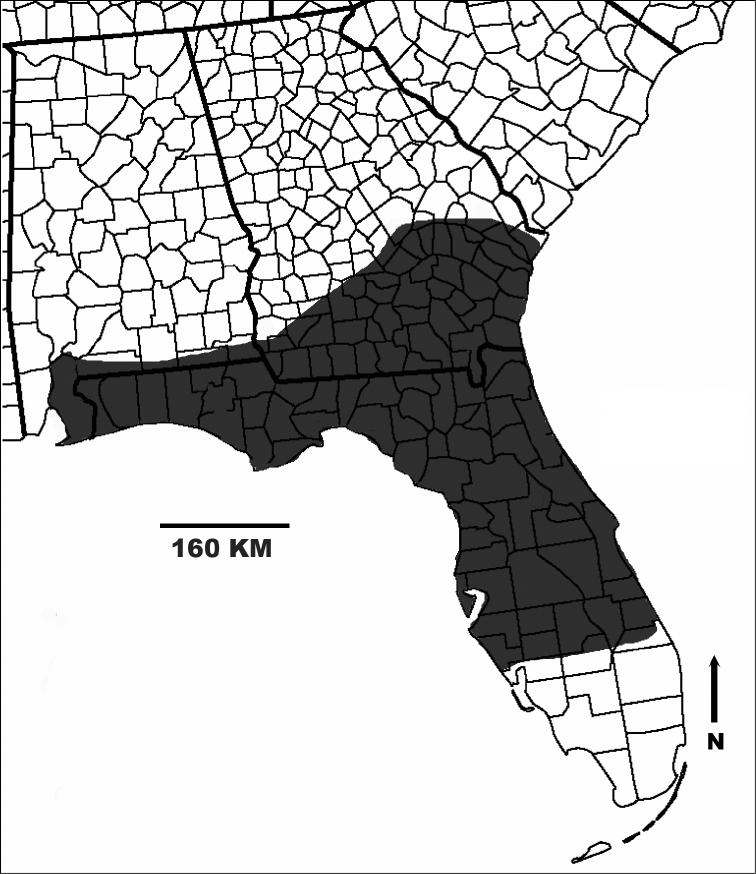
Geographic distribution of *Gymnoscirtetes*.

*Gymnoscirtetes*Scudder 1897 (Orthoptera: Acrididae) (Fig. 1.) is endemic to the southeastern portion of the North American Coastal Plain (Fig. 2). Scudder (1897) established the genus by describing *G.pusillus*. Hebard (1918) described a second species, *G.morsei*. Since then, no other taxonomic work has been conducted on the genus. These tiny, slender grasshoppers are inhabitants of low, moist, open portions of flatwoods, particularly when such areas slope to and border a bayhead, bog, fen, hydric hammock, swamp, or seasonal pond. Occasionally they can be found in grassy sandhills. They can often be found among dense patches of grass or other tall slender vegetation, where their gracile form and lateral striping provide effective camouflage.

*Gymnoscirtetes* is ideal for revisionary research. Their small size, inability to fly due to vestigial wings, habitat specialization, and disjunct distributions, combined with the high number of other endemic arthropods in the region, make it likely that new species await discovery. Also, male genitalia are typically used to delineate species in the subfamily and species established based on genitalia have been later supported by genetic analysis (Hubbell 1932; Otte 2012, 2014; Hill 2015; Huang et al. 2020). However, male genitalia have not been examined for this genus.

## ﻿Materials and methods

Most specimens examined in this study were collected by Dr. Theodore Hubble and Dr. Irving Cantrall of the University of Michigan Museum of Zoology (**UMMZ**), who made extensive collections of the genus with intentions to carry out a revision. However, no such study was ever completed. Other specimens examined in this study were borrowed from the Academy of Natural Sciences of Philadelphia (**ANSP**), Auburn University Museum of Natural History (**AUMNH**), the Florida State Collection of Arthropods (FSCA), the Mississippi Entomological Museum (**MEM**), and the United States National Museum (**USNM**). All type specimens of newly described species are deposited in the MEM, and paratypes will be deposited in ANSP, USNM, and UMMZ.

In order to conduct a thorough study of the genus, the male genitalia, which are typically concealed within the terminalia, were dissected and examined. Habitus and internal genitalia photographs were taken with a Leica Z16 stereoscope equipped with a Leica DFC420 camera at different stages during dissection. Images were automontaged with the Leica Application Suite. For scanning electron micrographs, specimens were mounted on stubs with silver paste and coated with 30 nm of platinum, then imaged with a JEOL–JSM65600F SEM. Measurements were made with a reticle mounted inside a Leica MZ12.5 stereomicroscope in the following ways:

Body Length — Dorsally from the fastigium vertices to the distal end of the genicular lobe of caudal femur in a parallel plane with the abdomen.

Pronotum length — Dorsally, along the median carina.

Cercus Length — Laterally, maximum possible measurement of the left cercus.

Cercus Basal Width — Laterally, along the point of attachment from the dorsal to ventral margin.

Mid Cercus Width — Laterally, at the mid-length of the left cercus.

Cercus Apex Width — Laterally, along the distal end.

Subgential Plate Tubercule Length — Laterally, from the base to the apex.

Subgential Plate Tubercule Width — Posteriorly, at the widest point.

## ﻿Results

Based on male morphology and distribution, *Gymnoscirtetes* easily divides into two distinct species groups. The *morsei* group comprises two species that are western in distribution, from Mobile Bay, Alabama, to the Ocklochnee River, Florida (Fig. 12). The *pusillus* group comprises four species that are eastern, from the Ocklochnee River, Florida (i.e., the eastern edge of the *morsei* group) to east Georgia, and south towards Lake Okeechobee, Florida.

### ﻿Checklist of groups and species

#### morsei *group*

**Figure 3.**
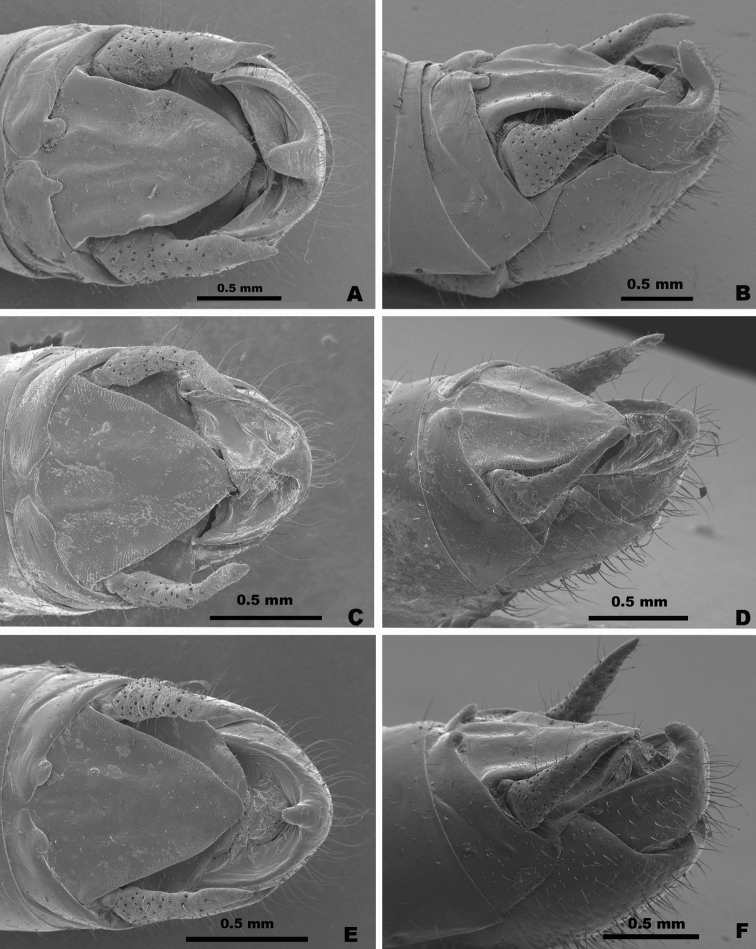
Dorsal and lateral SEM of *Gymnoscirtetes* male terminalia **A***G.morsei* (dorsal) **B***G.morsei* (lateral) **C***G.rex* (dorsal) **D***G.rex* (lateral) **E***G.pusillus* (dorsal) **F***G.pusillus* (lateral). Note: *G.pageae*, *G.wadeorum*, and *G.georgiaensis* are similar to *G.pusillus*.

**Figure 4.**
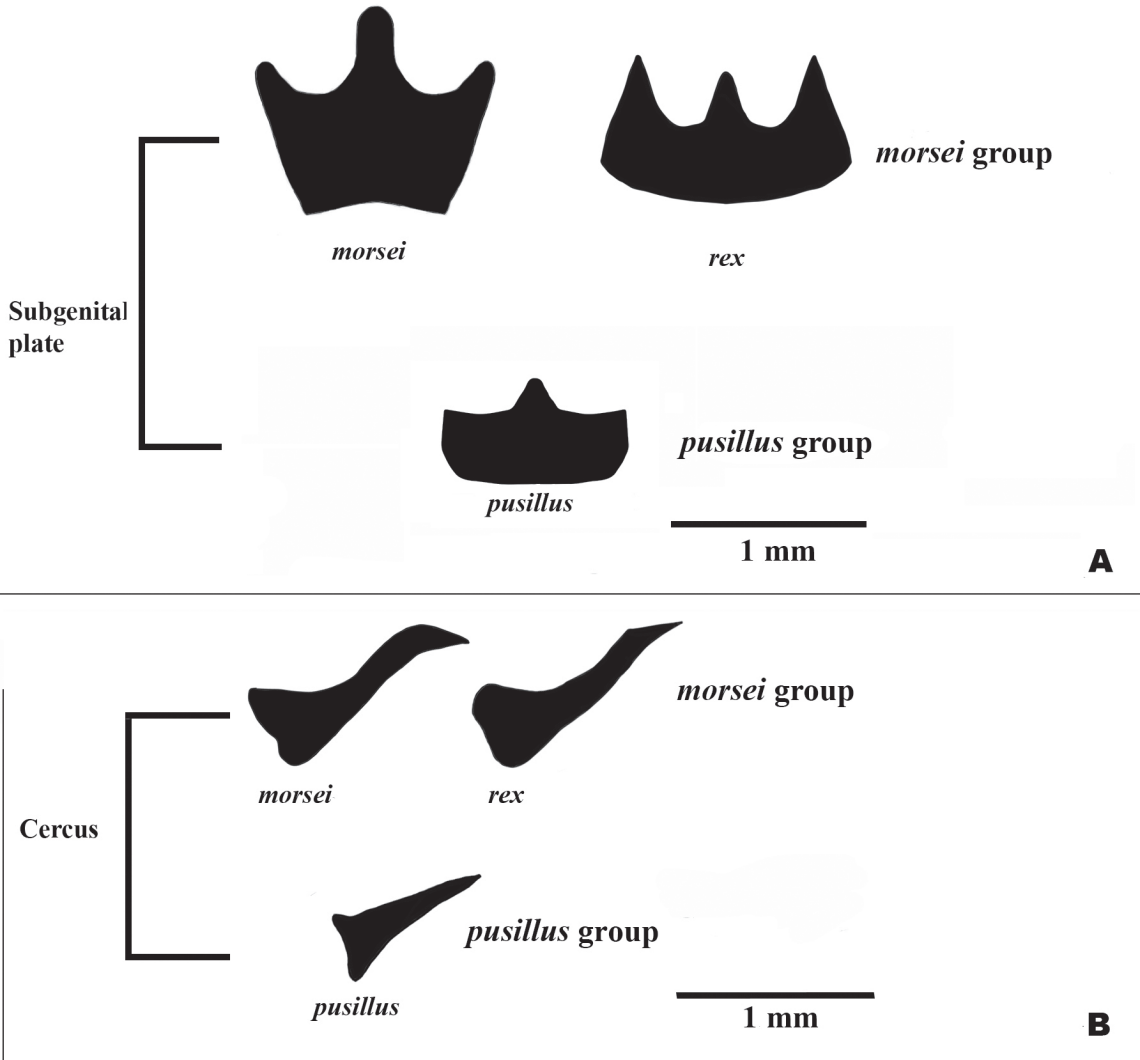
Partial silhouettes of *Gymnoscirtetes* male terminalia: **A** subgenital plate in caudal view **B** cerci in lateral view. Note: *G.pageae*, *G.wadeorum*, and *G.georgiaensis* are similar to *G.pusillus*. Within species groups there may be overlap in the shape of the cerci, and the shape is highly dependent on angle of view. Those pictured here are drawn from single individuals.

**Figure 5.**
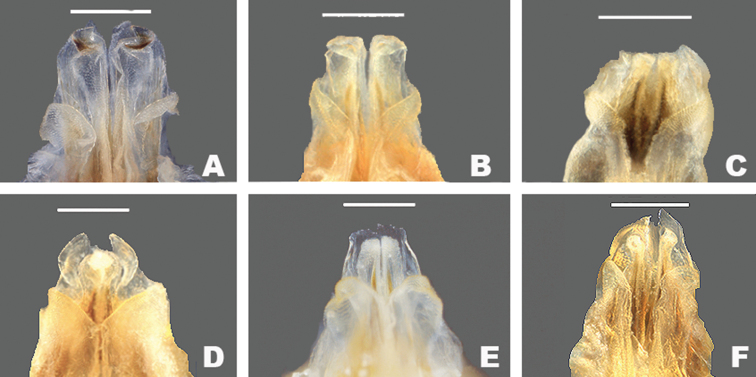
Caudal view of *Gymnoscirtetes* male aedeagi **A***G.morsei***B***G.rex***C***G.pusillus***D***G.pageae***E***G.wadeorum***F***G.georgiaensis*. Scale bars 0.2 mm.

**Figure 6.**
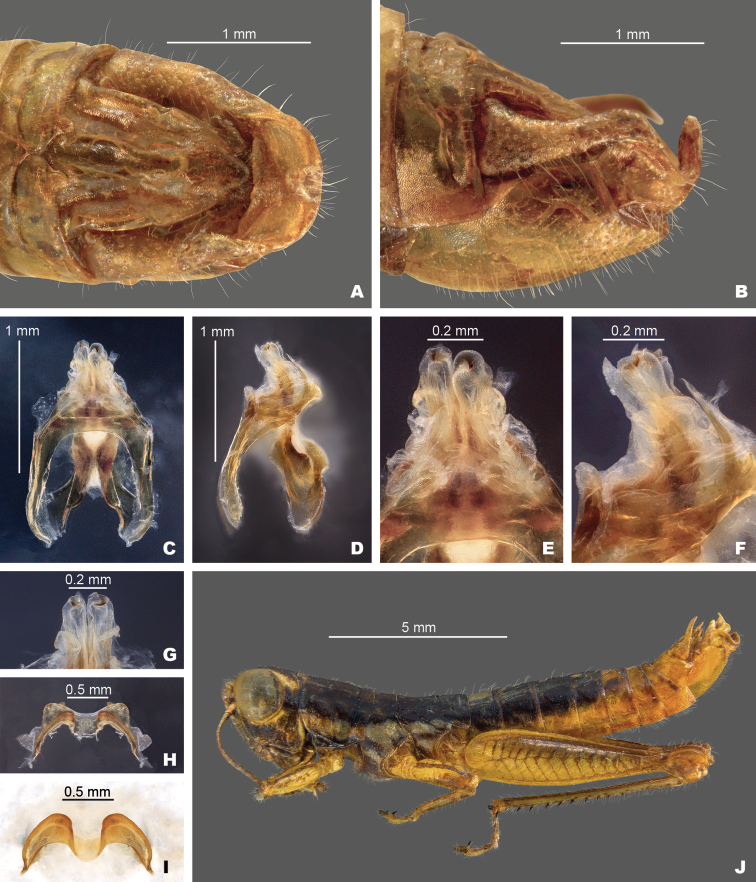
*Gymnoscirtetesmorsei*: **A** dorsal view of male terminalia **B** lateral view of male terminalia **C** dorsal view of phallic complex **D** lateral view of phallic complex **E** dorsal view of aedeagus **F** lateral view of aedeagus **G** caudal view of the aedeagus **H** dorsal view of epiphallus **I** caudal view of epiphallus **J** habitus.

**Figure 7.**
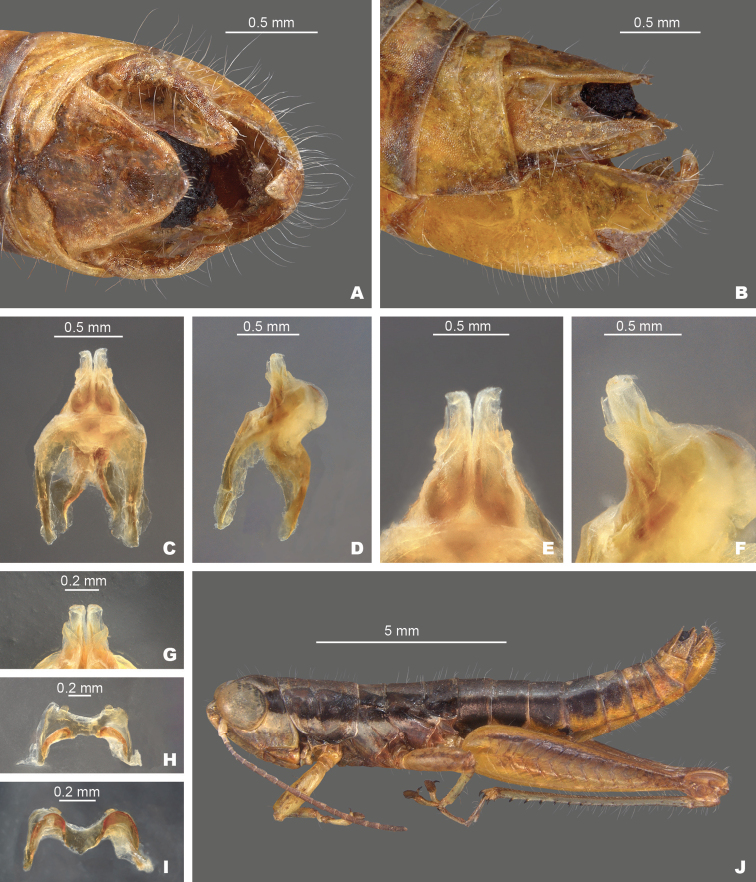
*Gymnoscirtetesrex*: **A** dorsal view of male terminalia **B** lateral view of male terminalia **C** dorsal view of phallic complex **D** lateral view of phallic complex **E** dorsal view of aedeagus **F** lateral view of aedeagus **G** caudal view of the aedeagus **H** dorsal view of epiphallus **I** caudal view of epiphallus **J** habitus.

1. *Gymnoscirtetesmorsei*Hebard 1918 — Figs 3A, B, 4A, 5A, 6A–K, 12, 14C

2. *Gymnoscirtetesrex* sp. nov. — Figs 3C, D, 4A, 5B, 7A–K, 12, 14E

#### pusillus *group*

**Figure 8.**
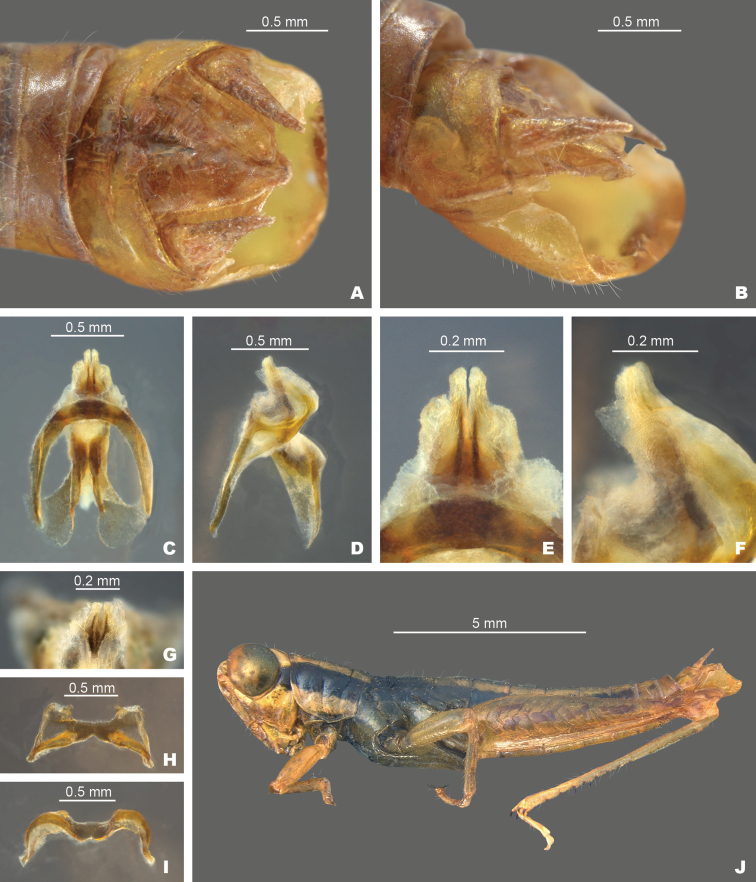
*Gymnoscirtetespusillus*: (type) **A** dorsal view of male terminalia **B** lateral view of male terminalia **C** dorsal view of phallic complex **D** lateral view of phallic complex **E** dorsal view of aedeagus **F** lateral view of aedeagus **G** caudal view of the aedeagus **H** dorsal view of epiphallus **I** caudal view of epiphallus **J** habitus.

**Figure 9.**
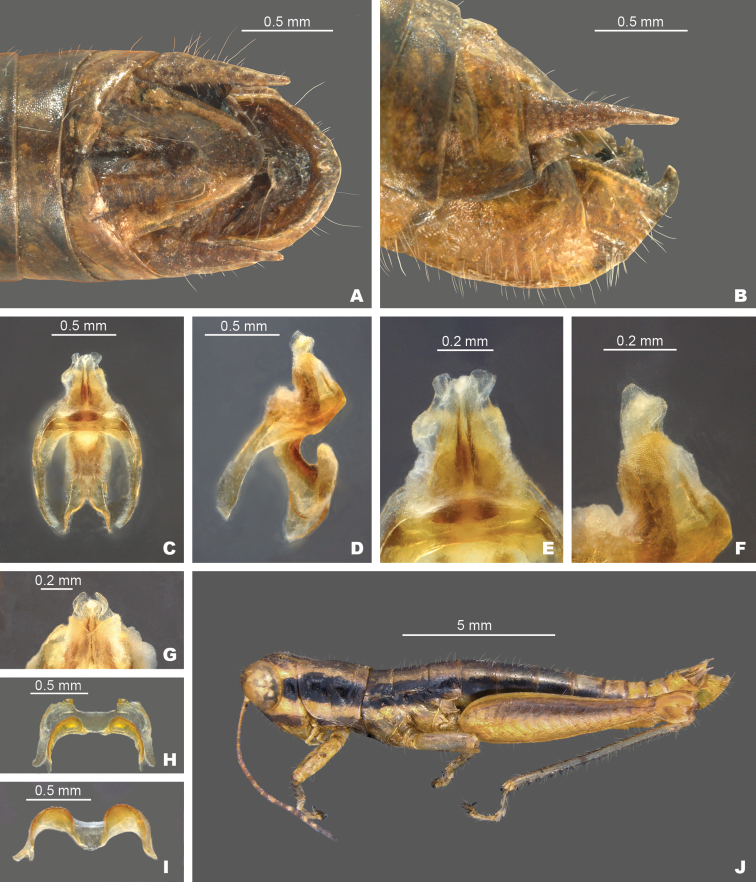
*Gymnoscirtetespageae*: **A** dorsal view of male terminalia **B** lateral view of male terminalia **C** dorsal view of phallic complex **D** lateral view of phallic complex **E** dorsal view of aedeagus **F** lateral view of aedeagus **G** caudal view of the aedeagus **H** dorsal view of epiphallus **I** caudal view of epiphallus **J** habitus.

**Figure 10.**
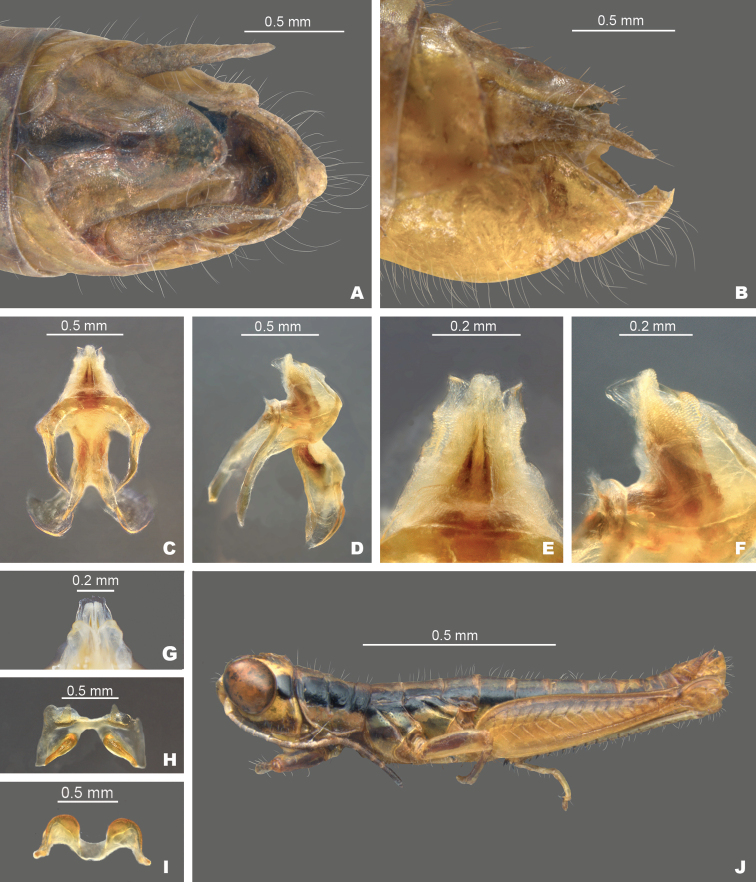
*Gymnoscirteteswadeorum*: **A** dorsal view of male terminalia **B** lateral view of male terminalia **C** dorsal view of phallic complex **D** lateral view of phallic complex **E** dorsal view of aedeagus **F** lateral view of aedeagus **G** caudal view of the aedeagus **H** dorsal view of epiphallus **I** caudal view of epiphallus **J** habitus.

**Figure 11.**
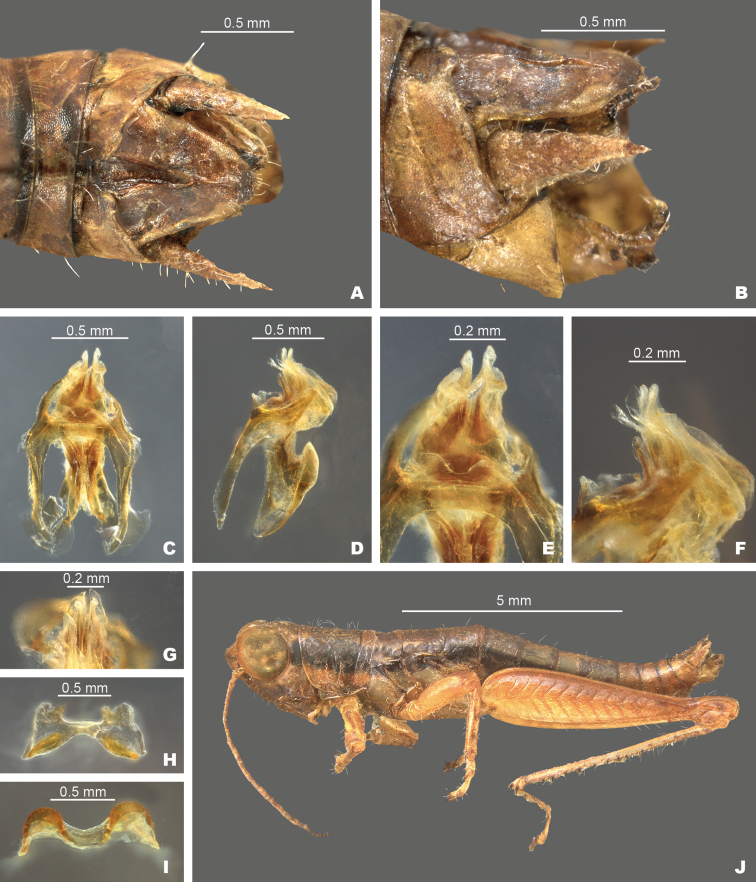
*Gymnoscirtetesgeorgiaensis*: **A** dorsal view of male terminalia **B** lateral view of male terminalia **C** dorsal view of phallic complex **D** lateral view of phallic complex **E** dorsal view of aedeagus **F** lateral view of aedeagus **G** caudal view of the aedeagus **H** dorsal view of epiphallus **I** caudal view of epiphallus **J** habitus.

**Figure 12.**
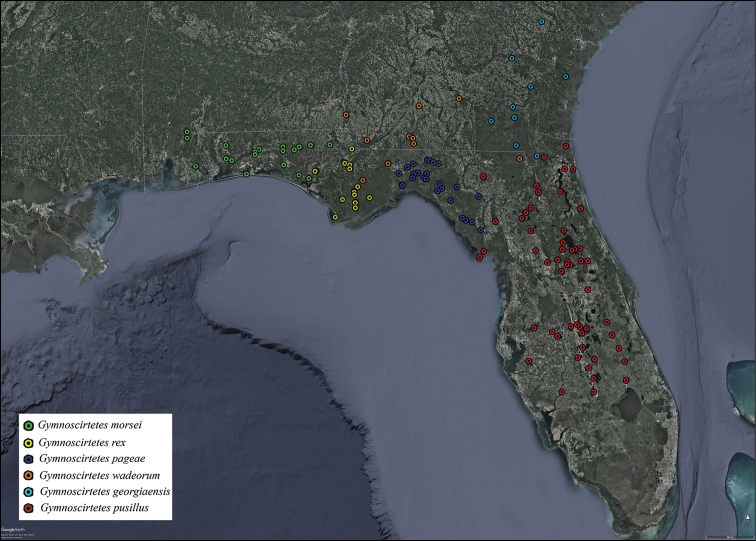
Distribution of *Gymnoscirtetes* species. Green dots = *G.morsei*, yellow dots = *G.rex*, dark blue dots = *G.pageae*, orange dots = *G.wadeorum*, Light blue dots = *G.georgiaensis*, red dots = *G.pusillus*.

3. *Gymnoscirtetespusillus*Scudder 1897 — Figs 3E, F, 4B, 5C, 8A–K, 12, 14H

4. *Gymnoscirtetespageae* sp. nov. — Figs 5D, 9A–K, 12, 14D, 14G

5. *Gymnoscirteteswadeorum* sp. nov. — Figs 5E, 10A–K, 12

6. *Gymnoscirtetesgeorgiaensis* sp. nov. — Figs 5F, 11A–K, 12

#### Comparison with related genera

##### Gymnoscirtetes

1. Small size (11–22 mm).
2. Body linear in shape (Fig. 1).
3. Appearing apterous with wings reduced to a minute, vestigial scale.
4. Body brownish-green or bronze with a black stripe running from behind the eye to near the end of the abdomen (Fig. 1).
5. Hind tibia and tarsi dull green.

##### Aptenopedes

1. Large size (17–28 mm).
2. Body somewhat elongate.
3. Wings developed into small linear pads.
4. Body green or brown with white and black striping.
5. Hind tibia blue, tarsi pink.

##### Eotettix

1. Size variable – small (10–20 mm) to larger (18–28 mm).
2. Body more robust.
3. Brachypterous but wings obvious.
4. Body green to bronze with a metallic luster; black postocular stripe.
5. Hind tibia and tarsi black or red depending on the species.

##### Floridacris

1. Larger (18–28 mm).
2. Body robust.
3. Wings reduced to small and slender pads.
4. Body green.
5. Hind tibia and tarsi pink.

##### Floritettix

1. Larger (16–29 mm).
2. Body robust.
3. Appearing apterous with wings reduced to a minute, vestigial scale.
4. Body green with black, white, or orange striping.
5. Hind tibia blue, tarsi pink.

#### Comparison of the species groups of *Gymnoscirtetes*

##### *morsei* group

1. Lateral lobes of subgenital plate expanded dorsally; tubercule longer than wide (Figs 3A–D, 4A).
2. Cerci generally falcate (Figs 3A–D, 4A).
3. Ventral valves of aedeagus more translucent; flattened (Figs 5A, B–7C–G).

##### *pusillus* group

1. Lateral lobes of subgenital plate not expanded; tubercule approximately as long as wide (Figs 3E, F, 4B).
2. Cerci triangular (Figs 3E, F, 4B).
3. Ventral valves of aedeagus more opaque; cylindrical (Figs 5C–F, 8–11C–G).

### ﻿Key to *Gymnosciritetes*

**Table:** 

1	Male cerci generally falcate with the apex nearly as wide as long at their bases (Figs 3A–D, 4A – *morsei* group); subgenital plate with lateral margins elevated as seen in caudal view (Fig. 4A – *morsei* group)	**2**
–	Male cerci generally triangular with the apex much narrower than the base (Figs 3E, F, 4B – *pusillus* group); subgenital plate without elevated lateral margins as seen in caudal view (Fig. 4B)	**3**
2	Tubercule of subgenital plate broader (Figs 3A, B, 4A); apex of cerci generally more falcate (Figs 3A, B, 4A); dorsal valves of the aedeagus more rounded (Figs 5A, 6C–G); western panhandle of Florida and extreme southern Alabama (Fig. 12)	***G.morsei***
–	Tubercule of subgenital plate narrower (Figs 3C, D, 4A), apex of cerci less falcate and sometimes rounded (Figs, 3C, D, 4B); dorsal valves of the aedeagus more truncate (Figs 5B, 7C–G); central to eastern portion of the Florida panhandle (Fig. 12)	***G.rex* sp. nov.**
3	Dorsal valves of the aedeagus shorter than the ventral valves (Figs 5C, 8C–G); north and peninsular Florida (Fig. 12)	***G.pusillus***
–	Dorsal valves of the aedeagus equal in length or nearly so to that of the ventral valves; not peninsular Florida	**4**
4	Dorsal valves of the male aedeagus rounded apically and expanded laterally such that they appear lobate (Figs 5D, 9C–G); “Big Bend” region of Florida (Fig. 12)	***G.pageae* sp. nov.**
–	Dorsal valves of the aedeagus truncated or slightly angular apically and not expanded laterally, usually parallel sided (Figs 5E, F, 10C–G, 11C–G); Florida and southern Georgia, but not the “Big Bend” region (Fig. 12)	**5**
5	Dorsal valves of the aedeagus more truncated apically and not twisted (Figs 5E, 10C–G); southern Georgia and northern Florida (Fig. 12)	***G.wadeorum* sp. nov.**
–	Dorsal valves of the aedeagus more angled apically (Fig. 5F), often with slight caudally directed twist (Fig. 11C–F); eastern Georgia (Fig. 12)	***G.georgiaensis* sp. nov.**

#### ﻿ Gymnoscirtetes

Taxon classificationAnimaliaOrthopteraAcrididae

Scudder 1987

01F19EF9-0063-5BF8-9A9E-0A0944731E66

Gymnoscirtetes Scudder, S.H. 1897. Proc. U.S. Nation. Mus. 20 (1124): 14

##### External morphology.

Species of small size (M: 11.8–17 mm, F: 17.5–22.2 mm). Body somewhat gracile and subcylindrical. ***Head*** slightly wider than pronotum; hypognathous with anterior margin of head steeply declivent; triangular dorsally. Fastigium broadening apically, and broadly concave. Eyes somewhat prominent, especially in males, and thinly separated by the narrow end of the fastigium. Antennae filiform, usually with 20–23 flagellomeres in males and 21–25 in females, but often 23–26; longer than the head and pronotum combined. ***Thorax*** with prosternal spine thin and subconical. Pronotum cylindrical, anterior margin sub-truncate, often somewhat emarginated, lateral margins parallel throughout, median carina either slightly indicated or obsolete, lateral carinae obsolete. Prozona 3–4 × as long as the metazona, anterior and median sulci present laterally but indistinct near the margins; prozona smooth and shiny. Metazona mostly smooth, but with occasional reticulations, posterior margin subtruncate. Lateral lobes of the pronotum declivent anteriorly and truncate posteriorly, the ventral posterior margin obtusely angulate. Wings vestigial, minute, scale-like. Metathoracic femur slender. Metathoracic tibia with 8–10 pairs of spines. Tympanal organ greatly reduced, appearing as a small depression or slit. ***Terminalia*** with furcula (males) (Fig. 1) rounded protuberances, projecting either slightly or moderately beyond the end of the segment from which they originate; bases minutely separated. Supra-anal plate (Fig. 1) triangular, slightly longer than wide, with the median groove anteriorly distinct with elevated sides, and diverging and becoming less distinct posteriorly. Cerci (Fig. 1) either short, triangular, tapering from base to apex or longer and subfalcate. Subgenital plate of male with a median tubercule (Fig. 1).

##### Phallic structures.

The dorsal valves are translucent to semi-translucent lobes that are flat, truncate, shortened to elongate depending on the species. The ventral valves are opaque and more strongly sclerotized than the dorsal valves, caudally projecting cylindrical lobes of various shapes depending on the species (Figs 1, 3, 4). The aedeagal sheath only covers the base of the valves (Fig. 2). The epiphallus is of the typical melanoploid shape, with lophi, ancorae, and an undivided bridge. But more precisely, the epiphallus of *Gymnoscirtetes* has a concave bridge, broadly rounded or arched lophi, convexly curved lateral plates that are sub-rectangular in shape with an angular anterior lobe and a short, rounded caudal tip, and ancora that are triangular, taper to a point, and are decurved ventrally.

##### Coloration.

Overall dull greenish brown to yellow, sometimes with bronze highlights. Antenna yellowish basally, remainder ferruginous. Antennal crescent complete. Head, thorax, and abdomen pale yellow, infuscated dorsally, especially along the midline. A lateral, well-defined, piceous, post-ocular stripe extends from the caudal margin of the eye through the thorax and towards the end of the abdomen; lateral area of head and thorax below post-ocular stripe creamy-yellow. Hind femora luteous. Hind tibia, pale dull green, often dulled basally; with black or black tipped spines (Figs 1, 4–9K).

##### Etymology.

*Gymno*, Greek, naked (in reference to the seemingly apterous condition); *skirtetes*, Greek, leaper.

##### Suggested common name.

Naked leapers.

***morsei* group**

**Diagnosis.** Typical of the genus but with male cerci generally falcate, subgenital plate with lateral lobes expanded dorsally, and central tubercle that is longer than wide (Fig. 3A–D). Ventral valves of aedeagus more translucent and not cylindrical in shape (Figs 4A, B, 6, 7C–G).

#### ﻿ Gymnoscirtetes morsei

Taxon classificationAnimaliaOrthopteraAcrididae

Hebard, 1918

47A494F3-A6E5-5651-8400-BC1A1F4E0678

Figs 3A, B , 4A , 5A , 6A–J , 12 , 14C

Gymnoscirtetes morsei Hebard, 1918: 142–143.

##### Diagnosis.

Most easily differentiated from the other species in the group based on the shape of the male cerci, which in *G.morsei* are decurved apically to an acute point (Fig. 6A, B), and by the shape of the male genitalia which have the dorsal valves rounded apically (Fig. 6G). The tubercle of the subgenital plate is often broader in most individuals of *G.morsei*, especially those in the western portion of the range.

##### Male measurements.

(mm): (*n* = 14) Body length 13.2–17.0 (mean = 14.6); pronotum length 1.9–2.6 (mean = 2.26); hind femur length 6.1–7.9 (mean = 6.9); cerci length 1.2–1.5 (mean = 1.3); basal width of cercus 0.4–0.7 (mean = 0.6); mid-cercal width 0.2–0.3 (mean = 0.2); cerci apex width 0.3–0.4 (mean = 0.4). tubercule length 0.3–0.4 (mean = 0.3); tubercule width 0.2–0.3 (mean = 0.2).

##### Female measurements.

(mm): (*n* = 7) Body length 19.5–21 (mean = 20.3); pronotum length 3.0–3.2 (mean = 3.1); hind femur length 9.0–9.8 (mean = 9.3).

##### Type information.

Florida, Walton County, Defuniak Springs, 30 August 1915, Rehn and Hebard (1♂).

##### Specimens examined.

**Alabama**, Baldwin County, 5.6 mi W Ala/Fla St. line on US 90, 13 September., 1954. T.H. Hubbell an I.J. Cantrall (14♂, 3♀); 6.2 mi SW Perdido on US Hwy 31, 13 Sept. 1954, T.H. Hubbell and I.J. Cantrall (1♂); Splinter Hill Bog, 31°01'30"N, -87°41'07"W, 19 July 2012, J.G. Hill, M.J. Thorn, Pitcher plant bog (1♂). **Florida**, Bay Co., 4.9 mi S Ebro, 16 October 1948, I.J. Cantrall (5♂), 9 mi E West Bay, 16 October 1948, I.J. Cantrall (1♂). Holmes Co., 0.4 mi E Ponce DeLeon, 14 September 1948, I.J. Cantrall (17♂); Westville, 23 August 1941 (7♂, 6♀); 0.6 mi E Bonifay, 14 October1948, I.J. Cantrall (6♂). Jackson Co., 1.4 mi W Cottondale 14 October 1948, I.J. Cantrall (1♂). Okaloosa Co., 3 mi E Crestview, 15 October 1949, I.J. Cantrall (3♂); 3.1 mi W Florosa, 15 October 1946. I.J. Cantrall (7♂). Santa Rosa Co., 2.3 mi S Junct. U.S. Hwy 90 and Hwy 87, 15 October 1949, I.J. Cantrall (4♂); 4.4 mi S Whitfields, 21 August 1951, I.J. Cantrall (7♂, 5♀); Milton, 15 August 1955, I.J. Cantrall (1♂). Walton Co., 2.3 mi N Freeport, 15 October 1948, I.J. Cantrall (1♂). 3.8 mi N Defuniak Springs, 14 October 1948, I.J. Cantrall (14♂).

##### Distribution.

Mobile Bay (Baldwin County, AL) east through the panhandle of Florida to Bay and Jackson counties (Fig. 12).

##### Habitat.

**Figure 13.**
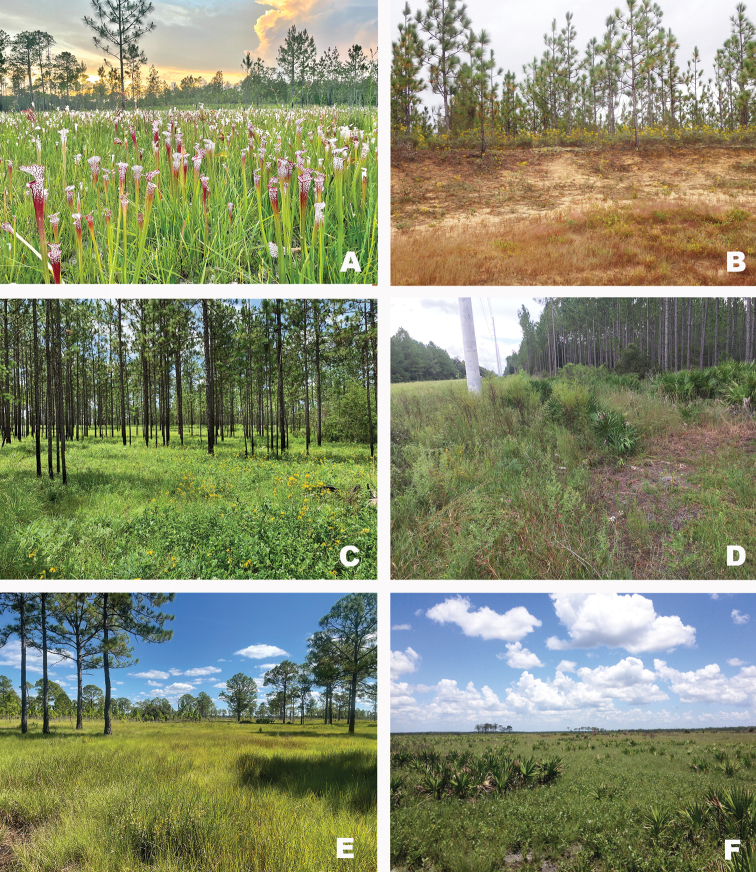
Plant communities at the collection localities of *Gymnoscirtetes***A** pitcher plant bog (Splinter Hill Bog, Baldwin Co., Alabama) **B** Sandhill (Ecofina Creek Wildlife Management Area, Bay County, Florida) **C** long leaf pine savanna (Wade Tract, Thomas County, Georgia) **D** Mesic sandhill (6 mi S Old Town, Dixie County, Florida) **E** Cutthroat grass seep (Royce, Highlands County, Florida) **F** Florida dry prairie (Avon Park Air Force Range, Highlands County, Florida).

Hebard (1918) describes the type locality at De Funiak Springs, Florida as being “a boggy area of wire-grass and bog plants, which was not more than fifteen yards wide by forty yards long”. At Splinter Hill Bog, in Baldwin County, AL (Fig. 13A), *G.morsei* occurs in a large bog dominated by *Sarracenialeucophyllia* Raf. and other carnivorous plants.

#### ﻿ Gymnoscirtetes rex sp. nov.

Taxon classificationAnimaliaOrthopteraAcrididae

7D668929-EF36-50CE-AAB0-136A0D39D248

https://zoobank.org/F8AB88FD-CAFF-4317-BE60-F4E099DD46B9

**Figure 14.**
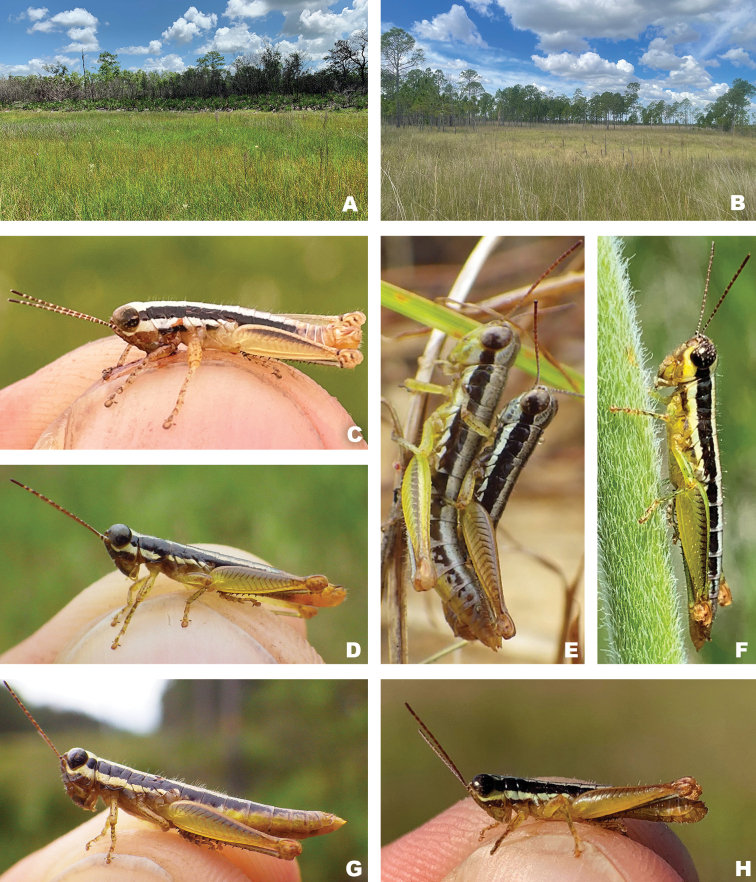
**A** Seasonal wetland (Lake Wales Ridge National Wildlife Refuge, Polk County, Florida) **B** seasonal wetland (Disney Wilderness Preserve, Osceola County, Florida) **C***Gymnoscirtetesmorsei*, male **D***Gymnoscirtetespageae*, male **E***Gymnoscirtetesrex*, pair in copula **F***Gymnoscirtetespusillus*, female **G***Gymnoscirtetespageae*, male **H***Gymnoscirtetespusillus*, male.

Figs 3C, D , 4A , 5B , 7A–J , 12 , 14H

##### Diagnosis.

Differs from *G.morsei* in having more narrow male cerci and curving or rotating medially apically, with the apex curving back laterally. In some individuals the apex of the cerci may be less acute or sometimes rounded (Fig. 7A, B). The dorsal valves of the male genitalia are truncated and decurved distally. The ventral valves are decurved and taper to a point distally (Fig. 7G). The tubercle of the subgenital plate is often narrower in most individuals of *G.rex* than in specimens of *G.morsei*.

##### Male measurements.

(mm): (*n* = 14) Body length 13.3–16.5 (mean = 14.7); pronotum length 2.3–2.5 (mean = 2.3); hind femur length 6.9–8.3 (mean = 7.5); cerci length 1.0–1.2 (mean = 1.1); basal width of cercus 0.4–0.5 (mean = 0.4); mid-cercal width 0.2 (mean = 0.2); cerci apex width 0.3 (mean = 0.3). tubercule length 0.1–0.3 (mean = 0.2); tubercule width 0.1–0.3 (mean = 0.2).

##### Female measurements.

(mm): (*n* = 14) Body length 18.3–22.2 (mean = 20.7); pronotum length 2.4–3.4 (mean = 2.9); hind femur length 8.5–10.0 (mean = 9.5).

##### Type information.

1♂, FLA., Bay Co., Ecofina Creek WMA, 30°25'41"N, -85°35'32"W, 27 October 2015, J.G. Hill, sandhill in short grasses and *Licaniamichauxii* Prance (Chrysobalanaceae). Deposited in the Mississippi Entomological Museum.

##### Paratypes.

Ecofina Creek WMA, 30°25'41"N, -85°35'32"W, 27 October 2015, J.G. Hill, sandhill (4♂, 5♀).

##### Other specimens examined.

**Florida**: Bay Co. 10 mi W Youngstown, 30°25'40"N, -85°35'25"W, 13 Sept. 2013, J.G. Hill (4♂, 7♀); Calhoun, 3.5 mi N Blountstown, 22 August 1951, I.J. Cantrall (5♂, 3♀); Blountstown, 22 August 1951. I.J. Cantrall (13♂, 10♀); 3.5 mi S Altha, 22 August 1951, I.J. Cantrall (1♂). Franklin Co., 3.1 mi S Sumatra on Fla. 65, 23 August 1951, I. J. Cantrall (1♂); 8.3 mi S Sumatra on Fla 65, 23 August 1951, I.J. Cantrall (11♂, 4♀). Gulf Co., 2.2 mi S Port St. Joe, 16 October 1948, I.J. Cantrall (3♂); 6.8 mi S Wewahitchka, 16 September 1940, I.J. Cantrall (1♂). Jackson, 0.9 mi E Grand Ridge, 14 October 1948, I.J. Cantrall (1♂). Liberty Co., 3 mi S Wilma on Fla 65, 23 August 1951, I.J. Cantrall (13♂, 5♀); 3.2 mi N Wilma on Fla 65, 23 August 1951, I.J. Cantrall (12♂, 8♀); 4.3 mi N Sumatra on Fla 12, 23 August 1951, I.J. Cantrall (8♂, 7♀) 7.9 mi N Sumatra on FLA 12, 23 August 1951, I.J. Cantrall (4♂, 3♀); 14.1 mi W Sumatra, 23 Sumatra 1941, I.J. Cantrall (9♂, 14♀).

##### Distribution.

Occurs in a narrow portion of the eastern Florida panhandle. At present, it is known only from Bay, Calhoun, Franklin, Gulf, and Liberty counties (Fig. 12).

##### Etymology.

From the Latin *rex* for monarch, in reference to the crown-like shape of the subgenital plate. The inspiration for this name came one day while at a local coffee shop (929) that had a crown as part of their logo. The shop was selling crown-shaped cookies by the cash register. I was working on this revision at the time and the shape of the cookies instantly remined me of the shape of the subgenital plate of this species.

##### Habitat.

This species can be found in much drier conditions that other members of the genus. At the type locality this species inhabited fine grasses in a sandy upland with *Chrysomapauciflosculosa* (Michx.) Greene (Fig. 13B). I have also collected this species from a large expanse of *Quercusminima* (Sarg.) Small in a sandhill. Specimen notes from other specimens indicate it inhabits bogs and savannahs as well.

***pusillus* group**

**Diagnosis.** Typical of the genus, but with the male cerci triangular and subgenital plate with the lateral lobes not expanded dorsally and with the tubercule approximately as long as wide (Figs 3C, 4B). Ventral valves of the aedeagus opaque and cylindrical in shape (Figs 8–11A–G).

#### ﻿ Gymnoscirtetes pusillus

Taxon classificationAnimaliaOrthopteraAcrididae

Scudder, 1897

BCD1C277-3192-582E-96D8-D250DE201736

Figs 3E, F , 4B , 5C , 8A–J , 12 , 14G

Gymnoscirtetes pusillus Scudder, 1897: 15

##### Diagnosis.

Differs from other species in the group based the shape of the internal male genitalia. In dorsal view, the dorsal valves are lightly sclerotized and semi-translucent, have apices that are rounded to sub-truncate, and shorter than the ventral valves (Fig. 8C, E). In lateral view, the dorsal valves taper to their apices and the ventral valves extend slightly past the dorsal valves with apices that are rounded to slightly angular (Fig. 8B, D, G).

##### Male measurements.

(mm): (*n* = 33) Body length 11.8–15.6 (mean = 14.4); pronotum length 1.8–3.1 (mean = 2.2); hind femur length 6.3–8.3 (mean = 7.3); cerci length 0.7–1.0 (mean = 0.8); basal width of cercus 0.3–0.4 (mean = 0.3); mid-cercal width 0.2 (mean = 0.2); cerci apex width 0.1 (mean = 0.1). tubercule length 0.1–0.2 (mean = 0.2); tubercule width 0.1–0.2 (mean = 0.2).

##### Female measurements.

(mm): (*n* = 28) Body length 18.5–21.2 (mean = 19.8); pronotum length 2.6–13.1 (mean = 3.1); hind femur length 8.5–10.3 (mean = 9.2).

##### Type information.

Florida [Duval Co.,] Jacksonville. Aug. [18]85.

##### Specimens examined.

**Florida**: Alachua Co., Fairbanks, 27 June 1924, F.W. Walker (2 ♂); Gainesville, 8 August 1925, T.H. Hubbell (4♂, 4♀); Same data as previous, except May 1926’ Waldo, 13 August 1924, F.W. Walker (4♂, 6♀). Same data as previous, except M.J. Thorn (2♂, 3♀); Bradford Co., 3.5 mi NE Lawtey, 1 August 1938, Hubbell and Friauf (1♂). Clay Co., Green Cove Springs, 30 September 1925, T.H. Hubbell (1♂). Duval Co., San Pablo (1♂). Gilchrist Co., 5 mi E Trenton, 14 August 1947, T.H. Hubbell (3♂). Highlands Co., Archbold B.S., 27.1813, -81.3545, 3 October 2021, M.J. Thorn (1♂, 1♀); Avon Park AFB, 27°38'12"N, -81°18'36"W, 16 June 2015, J. Hill, J. Barone, R. Noss (2♂, 3♀); Lake Wales Ridge NWR, 27.5153, -81.4130, 18 June 2019, J.G. Hill (5♀), Lake Wales Ridge WEA, 27. 3712, -81.3412, 4 October 2021, J.G. Hill (2♂, 2♀). Hillsborough Co., 4 mi NE Thonotosassa, 18 August 1938, Hubbell and Friauf (1♂); Little Mantee River U.S. Hwy 41, Hubbell and Friauf (6♂, 3♀). Lake Co., .1 mi E Altoona, 25 July 1938, Hubbell and Friauf (6♂). Lake Co., 0.7 mi S Pitman, 27 August 1938, Hubbell and Friauf (1♂); 1.5 mi W Astor, 24 July 1938, Hubbell and Friauf (3♂); 1.5 mi E Lisbon, 24 August 1926, Hubbell and Friauf (2♂); 3.3 mi E Altoona, 28 August, Hubbell-Friauf (1♂). Levy Co., Cedar Key, 29 Sept. 1923, T.H. Hubbell (2♂); Sumner, 18 October 1924, T.H. Hubbell (3♂). Marion Co., Lake Weir, 27 August 1927 (2♂, 3♀); Ocala, 17 August 1935, (2♂, 4♀). Ocala Nat’l Forest, T17S, R26E, Sec 3, Hubbell and Friauf (6♂, 3♀), Ocala Nat’l Forest, Juniper Springs, 9 June 1938, Hubbell and Friauf (1♂), Ocala N.F., 29.2757, -81.6898, 16 June 2019, J.G. Hill (4♂, 4♀); 2.5 mi W Crow’s Bluff, 29 August 1938, Hubbell and Friauf (1♂). Nassau Co., 1 mi W O’Neil, 19 August 1947, T.H. Hubble, (1♂); 1.6 mi SW Crawford, 19 October 1941, T.H. Hubbell (1♂). Okeechobee Co. 5.6 mi S. Co. line on US 441, 27 August 1951, I.J. Cantrall (3♂, 4♀); 4.3 mi N. Okeechobee, 27 August 1951, I.J. Cantrall (3♂, 3♀). Orange Co., Winter Park, 26 August 1937 (1♂, 4♀). Osceola Co., 9.2 mi S Kenansville, 27 August 1951, I.J. Cantrall (2♂, 3♀); 13.3 mi S Holopaw, 24 August 1951, I.J. Cantrall (23♂, 18♀); Disney Wilderness Pres. 28°04'06"N, -81°24'25"W, 17 June 2015, J.G. Hill, J.A. Barone (3♀); Holopaw, 27 August 1925, T.H. Hubbell (3♂). Pasco Co., Tribley (1♂). Polk Co., Haines City, 27 August 1925, T.H. Hubbell (1♂); Lake Streety, T 32S, R27S, Sec. 25, 10 August 1938, Hubbell and Friauf (1♂); Hatchineha Ranch, 28.008, -81.4839, 3 October 2022, J.G. Hill (1♂, 1♀); Lakeland, 28 June 1935, I.J. Cantrall (2♂); Lake Marion Creek WMA, 28.0992, -81.5121, 3 October 2021, J.G. Hill (1♂, 1♀); Lake Wales Ridge NWR, 28.1308, -81.5530, 3 October 2021, J.G. Hill (1♂, 1♀); Tiger Creek NA, 27°48'32"N, -81°29'24"W, 17 June 2015, J.G. Hill, J.A. Barone (2♂, 6♀). Putnam Co., Mannville, 22 Nov. 1938, T.H. Hubbell (1♂); Welaka, 21 August 1940, J.J. Friauf (1♂); same data as previous, except 8 August 1939 (1♂). St. Johns Co., 1.3 mi E jct US 1 and FLA 206, 26 August 1951, I.J. Cantrall (6♂, 8♀); Saint Augustine, 6 July 1935, I.J. Cantrall (2♂, 1♀). Suwannee Co., Houston, 23 August 1925. T.H. Hubbell (5♂). Volusia Co., 1.6 mi E Astor, 29.1667, -81.5000, 3 June 2021, J.G Hill and M.J. Thorn (3♂); 0.6 mi W Barberville, 6 Sept. 1938, Hubbell and Friauf (3♂).

##### Distribution.

Peninsular Florida from the northeast boarder with Georgia along the Atlantic Ocean west to eastern bank of the Suwannee River and south to the southern borders of De Soto, Highlands, and Okeechobee Counties (Fig. 12).

##### Habitat.

Found in a variety of grassland situations from seasonal ponds (Fig. 14A), cutthroat grass seeps (Fig. 13E), and flatwoods on the Lake Wales Ridge to Florida dry prairies (Fig. 13F). Irvin Cantrall reports collecting this species in saltwater flats with *Juncus* and *Batismaritima* (Cantrall 1951, field notes).

#### ﻿ Gymnoscirtetes pageae sp. nov.

Taxon classificationAnimaliaOrthopteraAcrididae

60B10979-8537-56C2-AA4C-A59A69E29787

https://zoobank.org/3F1242B8-CF10-4AFE-89DD-7DA9E0ECD0F2

Figs 5D , 9A–J , 12 , 14D, G

##### Diagnosis.

Differing from other species in the group based on the shape of the internal male genitalia (Fig. 9C–I). In dorsal view, the dorsal valves form two slightly translucent lobes that are equal to or are slightly longer than the ventral valves and are truncated apically. The ventral valves are opaque short cylindrical protrusions that are rounded at their apices. In lateral view, the dorsal valves are much broader than the ventral valves (~ 1.5 ×) and extend laterally up to or just short of the apex of the ventral valves. In caudal view, the dorsal valves form a girdle that almost completely encompasses the ventral valves like a hood (Fig. 9G). This species is perhaps the most distinct in the group and can readily be identified based on its unique genitalia and distinct geographic distribution (Fig. 12).

##### Male measurements.

(mm): (*n* = 14) Body length 13.5–16.9 (mean = 14.6); pronotum length 2.1–2.5 (mean = 2.3); hind femur length 6.7–8.3 (mean = 7.4); cerci length 0.8–1.1 (mean = 1.0); basal width of cercus 0.3–0.4 (mean = 0.4); mid-cercal width 0.2 (mean = 0.2); cerci apex width 0.1 (mean = 0.1) tubercule length 0.1–0.2 (mean = 0.2); tubercule width 0.1–0.2 (mean = 0.2).

##### Female measurements.

(mm): (*n* = 6) Body length 19.5–20.6 (mean = 20.0); pronotum length 2.5–3.0 (mean = 2.8); hind femur 8.5–9.6 (mean = 9.2).

##### Holotype.

6 mi S Old Town, 29.5156769, -83.0002496, 28 Sept. 2017, J.G. Hill, Collected from roadside sandhill and ditch (1♂). Deposited in the Mississippi Entomological Museum.

##### Paratypes.

Same data as type (2♂, 2♀).

##### Other specimens examined.

Florida: Dixie Co., 4 mi N Shamrock, 14 August 1947, T.H. Hubbell (1♂); 6 mi S. Steinhatchee R[iver], 5 August 1925, T.H. Hubbell (4♂); Cross City, 21 November 1925, T.H. Hubbell (1♂). Jefferson Co., 0.4 mi N Lamont, 16 August, 1947, T.H. Hubbell (5♂); 0.4 mi NE Fanlew, 16 August 1947, T.H. Hubbell (1♂); 0.7 mi N Jct. US 90 and Fla 257, 17 August 1947, T.H. Hubbell (1♂); 0.9 mi E Thomas City, 16 August, 1947, T.H. Hubbell (1♂); 4.4 mi NE Fanlew, 16 August 1947, T.H. Hubbell (1♂); 4.6 mi E Monticello, 17 August 1947 (1♂); near Covington, 31 Oct, 1942, T.H. Hubbell (5♂); Lloyd, 20 August 1938 (2♂, 4♀). Lafayette Co., 2 mi W Taylor County Line, 9 June 1941, Friauf and Hubbell (1♂); 12 mi W Mayo, 9 November 1941, Friauf & Hubbell (1♂). Leon Co., Chaires, 4 August1925, T.H. Hubbell (4♂). Taylor Co., 4.7 mi N Salem, 7 October 1945, T.H. Hubbell (1♂); Boyd, 15 October 1942, T.H. Hubbell (1♂); Perry, 5 August 1925, T.H. Hubbell (2♂); Hampton Springs, 31 October 1947, T.H. Hubbell (2♂). Madison Co. 2 mi E Aucilla River on US 90, 17 August 1947, T.H. Hubbell (1♂); 1.7 mi N Shady Creek, 16 Sept. 1942, T.H. Hubbell (1♂). Wakulla Co., 1.5 mi NW St. Marks, 15 August 1947, T.H. Hubbell (2♂).

##### Distribution.

“Big Bend” region of Florida from Leon and Wakulla counties, south through the flatwoods to the western banks of the Suwannee River in Dixie County (Fig. 12).

##### Habitat.

Flatwoods and grassy sandhills (Fig. 13D).

##### Etymology.

Named in honor of Bettie Mae Page, an iconic American photo model and former resident of Florida, who rose from a background of poverty and abuse to become a symbol of self-expression and body positivity.

#### ﻿ Gymnoscirtetes wadeorum sp. nov.

Taxon classificationAnimaliaOrthopteraAcrididae

6A88662B-6BC1-5606-8B0A-F678F7EDF552

https://zoobank.org/E3ED601A-90E3-486B-9D8D-C74C5333D025

Figs 5E , 10A–J , 12

##### Diagnosis.

Differing from other species in the group based on the shape of the internal male genitalia (Fig. 10C–I). In dorsal view, the dorsal valves form two translucent lobes that are equal to or slightly longer than the ventral valves and are truncated apically. The ventral valves are opaque short cylindrical protrusions that are rounded at their apices. In lateral view, the dorsal valves are broader than the ventral valves and taper to their apices. In caudal view, the dorsal valves extend above the ventral valves (Fig. 10G). This species is very similar to *G.pusillus* but is distinguished from that species by the length and angle of the dorsal valves which are longer and are angled more dorsally in *G.wadeorum*, and their separate geographic distributions (Fig. 12).

##### Male measurements.

(mm): (*n* = 14) Body length 13.0–15.1 (mean = 14.1); pronotum length 1.8–2.3 (mean = 2.2); hind femur length 6.7–7.8 (mean = 7.0); cerci length 0.7–1.0 (mean = 0.9); basal width of cercus 0.3–0.4 (mean = 0.3); mid-cercal width 0.1–0.2 (mean = .02); cerci apex width 0.1 (mean = 0.1). tubercule length 0.1–0.2 (mean = .01); tubercule width 0.1–0.2 (mean = 0.1).

##### Female measurements.

(mm): (*n* = 9) Body length 18.5–21.8 (mean = 20.0); pronotum length 2.5–3.0 (mean = 2.8); hind femur length 8.6–9.6 (mean = 9.1).

##### Holotype.

GA., Thomas Co., Wade Tract, 30°45'35"N, -84°00'01"W, 4 August 2011, J.G. Hill; Old growth longleaf pine savanna (1♂). Deposited in the Mississippi Entomological Museum.

##### Paratypes.

Same data as type (6♀).

##### Other specimens examined.

**Georgia**: Berrien Co., 1.1 mi S Appling, 11 Aug 1947, T.H. Hubbell (4♂, 2♀). Colquitt Co., Doerun Nat. Area., 31°17'17"N, -83°53'03"W, 14 October 2010, J.G. Hill, longleaf pine savannah (2♂). Decatur Co., Silver Lake WMA, 30°49'44"N, -84°45'14"W, 27 August 2010, J.G. Hill (1♂). Early Co., Williams Bluff NA, 31°11'58"N, -85°04'43"W, 18 June 2011, J.G. Hill (2♂). Thomas Co., 4.3 mi N Metcalf, 30.7634, -83.9915, 8 September 2022, J.G. Hill, J.R. Fisher; Greenwood Plantation, 30°50'10"N, -84°00'40"W, 4 August 2011, J.G. Hill (1♂); Same data as above, except 26 August 2010 (2♂, 2♀); River Creek WMA, 30°51'40"N, -84°04'04"W, 27 August 2010, J.G. Hill, longleaf pine savannah (3♂, 3♀); Same data as above, except 30°51'35"N, -84°04'37"W, 4 August 2011 (1♂, 5♀); **Florida**: Baker Co., John Bethea State For. 30.4834, -82.3002, 2 June 2021, J.G. Hill (5♂, 5♀), Gasden Co., 2 mi N Ochlookonee, 14 October 1948, I.J. Cantrall (1♂). Liberty Co., 5.3 mi S Telogia on Fla 65, 23 August 1951, I.J. Cantrall (7♂, 13♀).

##### Distribution.

Found in southern Georgia and north Florida, from Berrien County, GA west to the Chattahoochee River, and south to Liberty and Baker Counties, FL (Figs 12, 13C).

##### Habitat.

Flatwoods and pitcher plant bogs. I observed this species feeding on *Seymeriacassioides* (J.F.Gmelin) S.F.Blake at Doerun pitcher plant bog.

##### Etymology.

Named in honor of the Wade Family who, in 1979, placed an 85-ha tract of old growth longleaf pine savanna into a perpetual conservation easement. Today, the “Wade Tract”, is one of the most important remaining examples of the long leaf pine ecosystem in existence and is also the type locality of this species.

#### ﻿ Gymnoscirtetes georgiaensis sp. nov.

Taxon classificationAnimaliaOrthopteraAcrididae

647F95F2-4AFF-52C0-8373-5F69D2CA8895

https://zoobank.org/58A5654C-C0D0-4DBE-8B85-9538DE377F5F

Figs 5F , 11A–J , 12

##### Diagnosis.

Differing from other species in the group based on the shape of the internal male genitalia (Fig. 11C–I). In dorsal view, the dorsal valves form two translucent lobes that are nearly equal in length to the ventral valves and are pointed at their apices. The ventral valves are opaque cylindrical protrusions that are pointed at their apices. In lateral view, the dorsal valves are nearly equal in length to the ventral valves, twist caudally and taper to their apices. In caudal view, the dorsal valves extend above the ventral valves (Fig. 11G). This species can most easily be separated from *G.pusillus* by having longer dorsal and more translucent dorsal valves and from *G.wadeorum* by the more angled apices and the slight caudal twist in the dorsal valves. *G.georgiaensis* can also be distinguished from these species by their separate geographic distributions (Fig. 12).

##### Male measurements.

(mm): (*n* = 8) Body length 11.5–14.0 (mean = 12.8); pronotum length 1.7–2.2 (mean = 1.9); hind femur length 5.8–7.2 (mean = 6.6); cerci length 0.7–0.9 (mean = 0.8); basal width of cercus 0.3–0.4 (mean = 0.3); mid-cercal width 0.1–0.2 (mean = 0.2); cerci apex width 0.1 (mean = 0.1) tubercule length 0.1–0.3 (mean = 0.2); tubercule width 0.1–0.3 (mean = 0.2).

##### Female measurements.

(mm): (*n* = 1) Body length 13.0; pronotum length 2.0; hind femur 6.6.

##### Holotype.

GA., Appling Co. Moody Forest N.A., 31°54'24"N, -82°18'46"W, 13 October 2010, J.G. Hill; open longleaf pine/wiregrass savannah, MEM 446532. (1♂) Deposited in the Mississippi Entomological Museum.

##### Paratypes.

Same data as type, except BOLD DNA JGH 0066, MEM 446531 (1♀).

##### Other specimens examined.

**Georgia**: Bullock Co. Lily Bog, 1 October 1983, D. Rymal, G. Folkerts (2♂). Charlton, St. George, 4 August 1939, Hubbell and Friauf (1♂). Clinch Co., Homerville, 27 August 1911, Rehn and Hebard (1♂). Ware Co., 10 mi S Waycross, Edge of Okefenokee Swp. 16 August 1964, Gurney (1♂); Okeefenokee Swamp, 30 July 1931, J.D Beamer (1♂). Waycross, 11 August 1903, A.P. Morse (1♂). Wayne Co., 1.8 mi N Screven, 19 October 1946. T.H. Hubbell (2♂); Jessup (1♂).

##### Distribution.

All known locations occur on the lower Coastal Plain of Georgia Bulloch County south to Ware and Charlton Counties (Fig. 12).

##### Habitat.

Flatwoods and pitcher plant bogs.

##### Etymology.

Named after the state of Georgia, from which this species is apparently endemic.

## ﻿Discussion

The four new species of *Gymnoscirtetes* described here further demonstrate the high levels of endemism and undiscovered biodiversity on the North American Coastal Plain. The apparent center of diversity for *Gymnoscirtetes* is the area along the northeast Gulf of Mexico where four of the six species are distributed. In this region the Apalachicola and Sewanee Rivers along with the Mobile/Tensaw River delta combined with ecological changes resulting from Pleistocene glacial cycles have produced important biogeographic barriers for isolating populations and generating new species resulting in a biodiversity hotspot with numerous terrestrial taxa that show similar patterns of divergence (Sorrie and Weakly 2001; Soltis et. al. 2006; Hill 2015; Huang et al. 2020).

A complete phylogeny of the North American Melanoplinae is under way but is still several years away from completion. As such, the origins of the five NACP endemic genera remain unknown. Until this work is completed, I hypothesize that *Gymnoscirtetes* arose from a western ancestor that spread into southeastern North America during the Miocene or Pliocene. *Gymnoscirtetes* species are inhabitants of several types of (often mesic) grasslands. Ancestral species could have spread eastward during periods of drier climatic conditions that favored the spread of grassland habitats. During the Miocene, a corridor of semiarid live oak-conifer woodlands, arid subtropic scrub, grassland, subdesert to desert vegetation existed on the Gulf Coastal Corridor (Axelrod 1958; Webb 1977; Albright 1998; Noss 2013). Arid plant conditions may not seem suitable for the spread of mesic grassland inhabiting species, but even under current conditions many of the mesic grasslands inhabited by *Gymnoscirtetes* are adjacent to or are interspersed among scrub environments. In some cases, they occur in grasslands that are only wet for a portion of the year as is the case in the hyper-seasonal Florida dry prairies. Populations of the ancestral species could have then repeatedly isolated by advancing and retreating glaciers during the Pleistocene, which would have also created larger rivers that would have acted as biogeographic barriers. The two species groups were probably isolated early on during the Pleistocene with each group radiating later during the period, as seen in the *Melanoplusscudderi* group (Huang et al. 2020).

Despite being relatively secure in terms of conservation at present, *Gymnoscirtetes* may be of conservation concern in the future. Many co-occurring plant communities (e.g., long leaf pine savannas and pitcher plant bogs) are imperiled and have undergone drastic reduction in the last 200 years. Thus, threats to *Gymnoscirtetes* are habitat loss from anthropogenic habitat alterations and potential loss of habitat from climate change which may result in the flooding of their low-lying environments near the edge of the Coastal Plain. Given the growing interest in the biodiversity of the North American Coastal Plain, and the recent classification of the region as a biodiversity hotspot, I hope that this study helps further conservation efforts in the region.

## Supplementary Material

XML Treatment for Gymnoscirtetes

XML Treatment for Gymnoscirtetes morsei

XML Treatment for Gymnoscirtetes rex

XML Treatment for Gymnoscirtetes pusillus

XML Treatment for Gymnoscirtetes pageae

XML Treatment for Gymnoscirtetes wadeorum

XML Treatment for Gymnoscirtetes georgiaensis
